# Functional Mechanisms and Roles of Adaptor Proteins in Abl-Regulated Cytoskeletal Actin Dynamics

**DOI:** 10.1155/2012/414913

**Published:** 2012-05-17

**Authors:** Mizuho Sato, Masahiro Maruoka, Tatsuo Takeya

**Affiliations:** ^1^Graduate School of Biological Sciences, Nara Institute of Science and Technology, 8916-5 Takayama, Ikoma, Nara 630-0192, Japan; ^2^Institute of Microbial Diseases, Osaka University, Osaka 565-0871, Japan; ^3^Laboratory of Single-Molecule Cell Biology, Tohoku University Graduate School of Life Sciences, Aoba-ku, Sendai, Miyagi 980-8578, Japan

## Abstract

Abl is a nonreceptor tyrosine kinase and plays an essential role in the modeling and remodeling of F-actin by transducing extracellular signals. Abl and its paralog, Arg, are unique among the tyrosine kinase family in that they contain an unusual extended C-terminal half consisting of multiple functional domains. This structural characteristic may underlie the role of Abl as a mediator of upstream signals to downstream signaling machineries involved in actin dynamics. Indeed, a group of SH3-containing accessory proteins, or adaptor proteins, have been identified that bind to a proline-rich domain of the C-terminal portion of Abl and modulate its kinase activity, substrate recognition, and intracellular localization. Moreover, the existence of signaling cascade and biological outcomes unique to each adaptor protein has been demonstrated. In this paper, we summarize functional roles and mechanisms of adaptor proteins in Abl-regulated actin dynamics, mainly focusing on a family of adaptor proteins, Abi. The mechanism of Abl's activation and downstream signaling mediated by Abi is described in comparison with those by another adaptor protein, Crk.

## 1. Introduction

The filamentous (F)-actin cytoskeleton is a fundamental component of all eukaryotic cells and plays an integral role in determining cell shape and locomotion. Thus, F-actin dynamics must be regulated strictly in a spatiotemporal manner for proper biological output. This is achieved through functional interaction with other cytoskeletal components, intermediate filaments (IFs) and microtubules (MTs), and hundreds of accessory proteins. Signaling factors that stimulate F-actin rearrangement such as extracellular matrices (ECMs) and growth factors are known to cause an increase in Abl tyrosine kinase activity, followed by the relocalization of Abl to specific F-actin structures such as focal adhesion sites, lamellipodia, and membrane ruffles depending on the stimulus [1, 2], implying a role for Abl in actin-based processes under the influence of upstream signals as well as downstream signaling machineries. Indeed, when the expression of Abl is downregulated or even the Abl kinase activity is suppressed, the dynamism of F-actin-based processes becomes impaired [3]. Therefore, tyrosine phosphorylation of specific substrates and the formation of actin cytoskeleton-remodeling protein complexes through Abl appear essential to the rapid and dynamic regulation of the assembly as well as disassembly of F-actin [4–7].

How can Abl be involved in such diverse aspects of actin dynamics? Primary as well as higher structural features may underlie such functional properties. Indeed, the overall domain structure of the Abl kinases is unique among the tyrosine kinase family [5, 6]. First, structural and biochemical analyses have revealed that multiple autoinhibitory mechanisms arise from interactions within the N-terminal domain structure [8–12]. The formation and disruption of this structure play a key role in the regulation of the kinase activity of Abl. Next, the Abl protein is also unique in that the kinase domain is followed by an unusual extended carboxyl (C)-terminal region which consists of multiple functional domains including an actin-binding region and so can directly create and respond to cytoskeletal changes that affect cell shape and movement [5]. Moreover, recent studies have revealed the functional role of adaptor proteins in regulation of the kinase activity and substrate recognition of Abl through the interaction with specific motifs in this portion, and the existence of the distinctive signaling cascade unique to each member in these processes also became evident, as described below [5, 6].

In this paper, we first introduce structural and functional characteristics of Abl, summarize the outline of Abl-mediated F-actin signaling cascades, and subsequently describe the roles and functional modes of an adaptor protein, Abi, in the process of Abl-mediated actin dynamics, in comparison with those of another adaptor protein, Crk.

## 2. Abl Tyrosine Kinase Family

### 2.1. Origin and Physiological Roles

The Abl gene was first isolated as a transforming gene in the Abelson murine leukemia virus (A-MuLV) [13], whose product, v-Abl, showed tyrosine kinase activity [14], and was subsequently determined to be an altered form of the cellular Abl gene (c-*abl*) [15]. In addition to the activation mechanism through incorporation into A-MuLV, a reciprocal t(9;22)(q34;q11)chromosomal translocation generates a chimeric Bcr-Abl protein, with elevated kinase activity, which has been frequently observed in chronic myeloid leukemia (CML) and adult acute lymphoblastic leukemia (ALL) patients [16, 17]. An Abl-related gene (or *arg*) is a paralog of c-*abl* identified by sequence similarity [18]. In addition to vertebrates, Abl-family genes have been found in *Drosophila melanogaster* (*dAbl*) and *Caenorhabditis elegans* (*ABL-1*) [19, 20].

Abl and Arg seem to have substantial functional overlap *in vivo*. Mice with a disrupted c-*abl* show defective development [21, 22] and responsiveness of B cells [23, 24] and T cells [25]. Some of the surviving c-*abl*−/− mice were reported to show osteoporosis, decreased systolic blood pressure, and cardiac hyperplasia [21, 22, 26]. In contrast, Arg's ablation leads only to relatively subtle neuronal defects [27]. The c-*abl*−/− *arg*−/− double mutation, however, causes embryonic death with abnormalities in neuroepithelial cells and defects in neurulation [27]. In addition, dysregulation of Abl leads to several pathological states; recent evidence suggests possible roles of Abl in breast-cancer invasiveness [28], neurological disorders [27], and microbial pathogenesis [29, 30].

### 2.2. Primary Structure and Functional Domains

**Figure 1:**
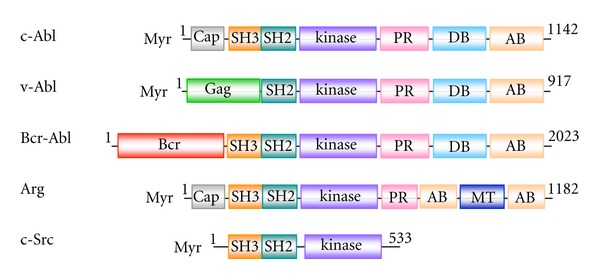
Primary structure of Abl kinases. Only isoform 1b of c-Abl and Arg in mice is shown. Both have a “Cap” region at the extreme N-terminus (Cap, gray), followed by the Src homology-3 domain (SH3, orange), Src homology-2 domain (SH2, blue-green), and catalytic domain (kinase, purple). However, at the extreme N-terminus, v-Abl contains a viral “Gag” sequence (Gag, green) by replacing the “Cap” and the SH3, and Bcr-Abl contains the N-terminal portion of the Bcr protein (Bcr, magenta) by replacing the “Cap,” which were generated by uptake into a retrovirus and chromosomal translocation, respectively. In the C-terminal half, Abl has four P-x-x-P motifs and Arg has three in the proline-rich region (PR, pink). At the extreme C-terminus, there are actin-binding domains in Abl and Arg (AB, beige). In addition, Abl has a DNA-binding region (DB, light blue), while Arg has a microtubule-binding domain (MT, blue). Amino acid residues of each gene in mice are numbered except for the human P210 Bcr-Abl gene [42]. The primary structure of c-Src is shown for comparison [43].

The overall domain structure of each Abl family protein is shown in Figure 1. The SH3, SH2, and kinase domains of c-Abl share only 52% identity, with 37% identity in the SH3 and SH2 domains, when compared to another nonreceptor tyrosine kinase, Src [6]. The “Cap” and SH3 domains are replaced with the viral “Gag” sequence in v-Abl [14], while the Abl breakpoint position in Bcr-Abl consistently leads to removal of the “Cap” sequence but retention of the SH3 domain [16]. In the C-terminal half, Abl and Arg contain proline-x-x-proline (P-x-x-P: x, any amino acid) motifs in the proline-rich region (PR) which allows interactions with SH3 domain-containing proteins and a calponin homology (CH) domain at its extreme C-terminus that binds to both F- and globular (G)-actin [31–33]. However, there is only 29% sequence identity between Abl and Arg in the C-terminal half [18], implying unique functions in each gene. Indeed, Abl contains a DNA-binding region [34, 35], nuclear localization signal motifs [35], and a nuclear export signal [36], allowing it to shuttle between the nucleus and cytosol [37]. Nuclear Abl plays a role in transcriptional regulation, particularly in response to DNA damage [38], and activation of the nuclear pool of Abl can induce apoptosis [39]. Cytoplasmic Abl, on the other hand, becomes localized to dynamic regions of the cytoskeleton including membrane ruffles, leading edges, and F-actin protrusions in response to extracellular stimuli such as growth factors like EGF and PDGF and cell adhesion [1, 7, 40]. The oncogenic Bcr-Abl and v-Abl proteins, however, do not enter the nucleus despite the presence of the nuclear localization signals [41]. 

## 3. Regulatory Mechanism of Abl Kinase Activity

**Figure 2:**
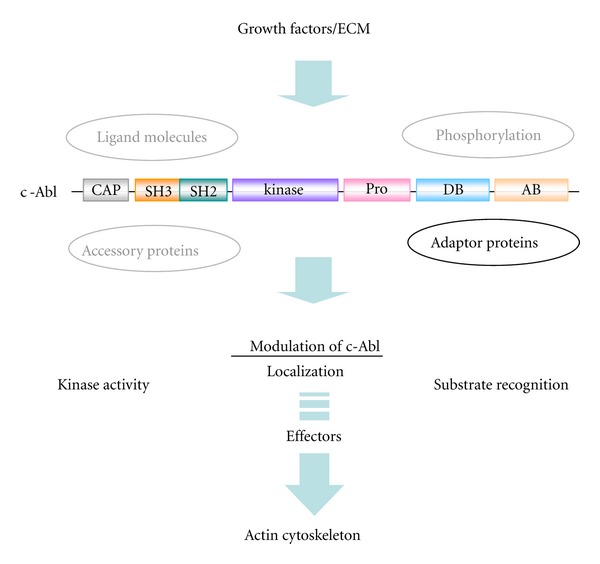
Signaling upstream and downstream of Abl leading to actin dynamics. Abl kinase activity, localization, and substrate phosphorylation which are responsible for F-actin dynamics may be modulated by ligand molecules, accessory proteins, phosphorylation by other kinases, and/or adaptor proteins as described in the text. However, the role of adaptor proteins is mainly highlighted in this paper. ECM: extracellular matrix.

Abl kinase activity is regulated in a variety of ways reflecting its domain structure, and this regulation may confer on Abl its role as a mediator of signaling in actin dynamics [4] (Figure 2). First, structural data and biochemical studies have revealed multiple autoinhibitory mechanisms that constrain the enzymatic activity. The N-terminal half consisting of the “Cap”, SH3, SH2, and kinase domains (residues 1–531 in c-Abl) represents the minimum segment for this autoregulation [8] in which interaction made by its SH3 and SH2 domains with the distal surface of the kinase domain imposes a “locked” inactive state [8, 9, 44–46]. Attachment of a myristoyl group to the N-terminus or phosphorylation in the “Cap” sequence further stabilizes this inactive conformation through additional surface interaction [8, 9]. Consequently, disruption of such structural hindrances leads to an increase in kinase activity as observed in v-Abl and Bcr-Abl (Figure 2). Release of the latch and clamp can also be achieved by binding of either the intracellular domain of activated integrins or P-x-x-P and/or phosphotyrosine-containing ligands to the SH3 and SH2 domains [5, 45]. Indeed, when fibroblasts were plated on fibronectin or treated with an *α*5 integrin cross-linking antibody, increased Abl kinase activity was observed [39]. However, full activation of c-Abl kinase activity requires the phosphorylation of both Y245 and Y412 [47–50]. Y245 resides between P242 and P249, which are responsible for the intramolecular interaction with the SH3 domain. The phosphorylation of Y245 can be achieved by the Src family kinases or autophosphorylation reaction in trans [48, 51] and is presumed to activate Abl kinase through the disruption of this intramolecular interaction, as has been observed in the P242E/P249E mutant [52]. Y412, on the other hand, is located in the activation loop of the kinase domain and conserved in all tyrosine kinases. Y412 has been identified as an autophosphorylation site accompanied by the elevation of intrinsic kinase activity and is presumed to be responsible for the binding of substrate [1, 6]. Indeed, such phosphorylated forms have been identified in the Bcr-Abl and v-Abl proteins [51, 53].

The importance of the C-terminal half in the regulation of Abl has been clarified in various studies [31–33]. For example, c-Abl mutant mice lacking the C-terminal portion but retaining the SH3, SH2, and kinase domains exhibit the same phenotype as *c-abl* knockout mice [22]. Moreover, adaptor proteins consisting of multi-SH3 domains have been identified to interact with the PR region of the C terminus in Abl/Arg (Table 1). Also, the binding of F-actin to the AB region could contribute by inhibiting the kinase activity [54]. Catalytic activity, on the other hand, is required for c-Abl to modulate the F-actin cytoskeleton, suggesting that there could be a bidirectional regulatory mechanism between Abl and F-actin in the process of F-actin recognition and modulation [4]. A contribution of the phosphorylation of Tyr and Ser residues in this region has also been proposed [6].

## 4. Regulation of Abl Kinase by Abi and Crk

### 4.1. Abi and Crk Families

Nine proteins have been identified in mice that bind the C-terminal half of Abl and function as adaptors (Table 1). Among them, the Abi and Crk families are known to be phosphorylated by Abl and, in turn, modulate Abl. Consequently, clarification of the mode of interaction between Abi/Crk and Abl has been a subject of intensive research in this field [59, 69, 70].

**Figure 3:**
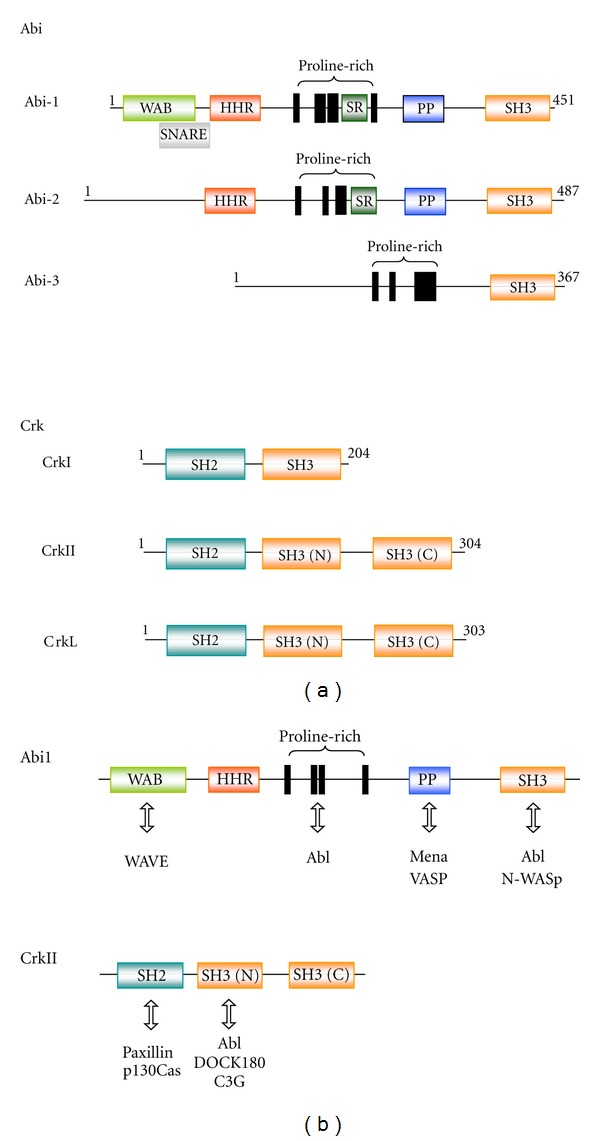
Domain structure of the Abi and Crk family proteins. (a) Modular domains of the Abi and Crk family proteins in mice are illustrated. For the Abi family, WAB (light green): WAVE-binding domain, SNARE (grey): Syntax-1 binding domain which is an overlapping domain of WAB, HHR (red): Hox homology region, SR: serine/threonine-rich region, PP (cyan): polyproline structure, SH3 (orange): SH3 domain [55, 71–75]. For the Crk family, SH2 (blue-green): SH2 domain, SH3 (orange): SH3 (N) and SH (C) domains [56, 57, 76]. Amino acid residues in each protein are numbered; only isoform 1 for Abi2 and Abi3 is shown. (b) Proteins reported to interact with Abi1 and CrkII involved in the regulation of Abl-mediated actin dynamics are shown.

The Abi family proteins, Abi1 and Abi2, were originally identified as proteins that bind to the P-x-x-P motif in Abl and negatively modulate its transforming activity [62, 77]. A third member, Abi3, was identified as a new gene in humans, NESH, possessing an SH3 domain, and was later incorporated into the Abi family on the basis of amino acid sequence similarity [78]. However, its binding to Abl has not been identified. Features of the primary structure of the Abi family are illustrated in Figure 3(a) [55–57, 71–76]; a proline-rich region and SH3 domain are common. c-Crk, on the other hand, is the cellular homolog of v-Crk which was isolated as a transforming gene of the CT10 retrovirus [55]. c-Crk is expressed as two distinct proteins, CrkI and CrkII, respectively, by an alternative splicing of c*-crk* mRNA [56, 57]. CrkI consists of one SH2 and one SH3 domain, while CrkII has an additional SH3 domain separated by a “spacer region” of approximately 60 amino acids: SH3 (N) and SH3 (C) (Figure 3(a)). The Crk SH2 domain shows affinity for the binding motif pY-x-x-P. Indeed, multiple motifs are present in prominent Crk SH2-binding proteins such as p130Cas and paxillin [69, 76]. A paralog of Crk, Crk-like (CrkL), has been identified [58]. CrkL also consists of one SH2 and two SH3 domains without any catalytic domain (Figure 3(a)) and interacts with the proline-rich region. Roles of CrkL have been mostly characterized in Bcr-Abl-positive cells [76, 79].

Abi1-null mice die in the midgestational stage and show phenotypes similar to those of *α*4 integrin or VCAM1 knockout mice, implying that Abi1 could be a key mediator of *α*4-mediated signaling at the leading edge [80, 81]. Abi1 was actually found to interact and colocalize at the leading edge of lamellipodia with phosphorylated *α*4, and deletion of Abi1 dramatically diminishes spreading of fibroblasts engineered to express *α*4 on both VCAM1 and fibronectin [81]. Mice lacking Abi2 showed abnormal phenotypes in the eye and brain [82]. On the other hand, Crk knockout mice exhibited marked growth retardation with poorly developed a heart and vasculature and died *in utero* [83]. These results imply distinctive roles for Abi1, Abi2, and Crk *in vivo*.

### 4.2. Regulatory Mechanism of Abl Kinase by Abi and Crk

**Figure 4:**
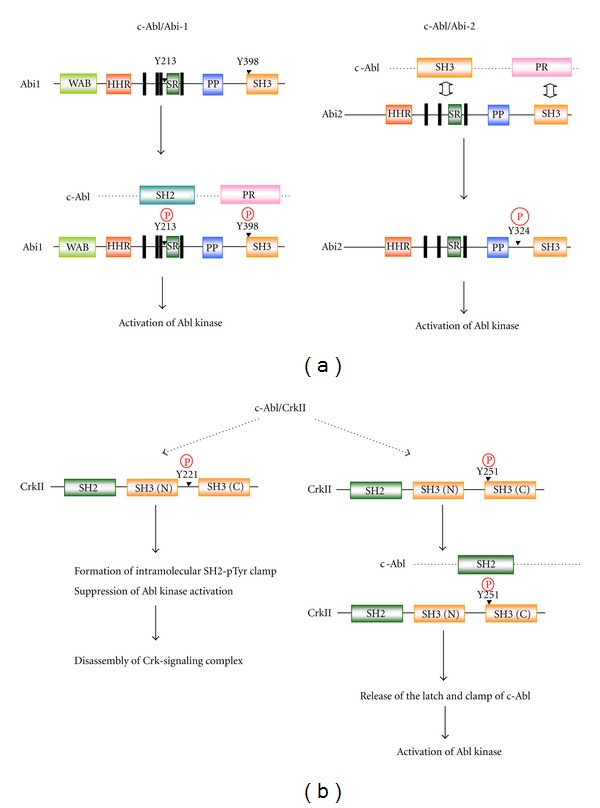
Regulatory mechanism of Abl kinase activity by Abi and Crk. (a) Events leading to the activation of the Abl kinase activity after the interaction of Abl and Abi1 or Abi2 are schematically illustrated. The regulatory Y213 and Y398 in Abi1 and Y324 in Abi2 are indicated. Phosphorylated Y213 and Y398 in Abi1 are involved in the interaction with c-Abl through the SH2 and PR region of c-Abl, respectively. (b) Y221 and Y251 in CrkII are sites of phosphorylation by Abl after the interaction of Abl and CrkII. When Y221 is phosphorylated, it induces an “autoinhibition” through an intramolecular SH2-pTyr clamp and the suppression of Abl kinase activity, resulting in the disassembly of Crk-mediated signaling complexes and abrogation of Crk-mediated signaling. On the other hand, Y251 in the SH3 domain interacts with the SH2 domain of Abl and releases the latch and clamp structure of Abl, leading to the activation of Abl.

Abi1 and Abi2 play a dual role as potential effectors and regulators of the Abl kinase [70, 84]. Abl is known to oligomerize through the N-terminal region (509 residues) depending on its kinase activity, and Abi1 also oligomerizes and an oligomeric form of Abi1 interacts with the Abl protein [85]. How the phosphorylation of Abi by Abl and/or autophosphorylation of Abl play roles in these steps remains to be elucidated. Regarding this, several tyrosine phosphorylation sites have been identified in Abi1 [86–90]. Among them, Y213 [89] and Y398 [90] were reported to play a role in the regulation of Abl (Figure 4(a)). Regarding this, preferred Abl target sites have been identified as I/V/L-Y-x_1-5_-P/F using an mRNA display method [91]. When compared, the flanking amino acid sequences of Y213 (D-Y-M-T-S-P) lacks an upstream hydrophobic residue, whereas Y398 (I-Y-D-Y-T-K-D-K) lacks a downstream proline/phenylalanine residue [92]. Phosphorylation of Y213 was reported to be involved in the binding to the Abl SH2 domain and consequently induce downregulation of its catalytic activity [89]. Phosphorylated Y213 was also shown to interact with the p85 C-terminal SH2 domain and negatively regulate p85-dependent macropinocytosis [93]. However, the interaction of a phosphopeptide with the SH2 domain induces a conformational change in Abl, increasing the kinase activity [45, 94]. Furthermore, phosphorylation of Y213 in Abi1 is seen in several Bcr-Abl-transformed leukemic cell lines [60]. Therefore, the significance of Y213 in the regulation of Abl awaits further clarification. Y398 in the SH3 domain of Abi1, on the other hand, was recently identified as another major site of phosphorylation by Abl [90]. Y398, located in the RT-loop of the SH3 domain, is highly conserved among other SH3 domain-containing proteins and presumed to play a role in the binding of target peptides [95]. Indeed, the SH3 domain of Abi1 and the proline-rich domain of Abl were identified as responsible for the interaction between Abi1 and Abl, leading to the activation of Abl kinase, whereas disruption of Y398, combined with Y213, significantly weakens the binding of Abi1 to Abl [90]. Abi2 binds Abl at two sites: one is through the SH3 domain of Abl and the P-x-x-P motif of Abi2, and the other is through the P-x-x-P motif of Abl and the Abi2 SH3 domain [62] (Figure 4(a)). Y324 of Abi2 is predicted as a site of phosphorylation by Abl based on a conserved flanking amino acid sequence (E-Y-S-D-P) [96] and is presumed to play a role in the regulation of Abl kinase activity [62]. However, since the Y213 and Y398 residues are also conserved in Abi2, pY213 and/or pY398 could play a role for Abi2 as in the case of Abi1.

The modulation of Abl kinase by Crk occurs in discrete steps [84, 97] (Figure 4(b)). The initial and an essential event is the interaction between the Crk SH3 (N) domain and three of the four PR motifs of Abl, inducing transactivation of c-Abl to phosphorylate CrkII at Y221 in the spacer region [98, 99]. This phosphorylation induces an intramolecular SH2-pTyr clamp and suppression of the c-Abl kinase activity as well, resulting in the disassembly of Crk-mediated signaling complexes and abrogates downstream signaling [100]. In contrast, v-Crk lacks Y221 and hence is presumed to take an open structure constitutively [97]. In addition to Y221, a recent study identified Y251 in the RT loop of SH3(C) as a second site of phosphorylation by Abl [101]. Y251, when phosphorylated, binds to the Abl SH2 domain to transactivate Abl, demonstrating a positive regulatory mechanism by CrkII. In this regard, functional significance as well as similarity between Y251 in the CrkII SH3(C) and Y398 in the Abi1 SH3 may be noted, although the target domain Abl seems to be different. A form of CrkII phosphorylated at Y251 has been observed in CML cell lines [101]. Tyrosine phosphorylation of Crk thus can both positively and negatively regulate Abl kinase activity and its signaling cascades (Figure 4(b)). The SH2 and SH3 (N) domains may be required for bridging other cellular proteins as substrates by Abl.

 The interaction of Abi and Crk with Abl is also regulated by the phosphorylation of Abl by other protein kinases. For example, the serine/threonine kinase Pak2 phosphorylates c-Abl at S637 and S638 which reside next to the P-x-x-P motif [102]. Phosphorylation at these sites weakens the binding of Abi1, while enhancing the binding of Crk, resulting in an increase in Abl kinase activity. Taken together, Abi1, Abi2, and Crk, as adaptor proteins, appear to regulate Abl in distinctive ways (Figure 4).

## 5. Signaling Cascades in Abl-Mediated F-Actin Dynamics in Normal and CML Cells and Involvement of Abi and Crk

### 5.1. Abl in Actin Dynamics

Abl transduces extracellular signals to cytoskeleton actin assembly/disassembly through its kinase activity. The actin-related protein-2/3 (Arp2/3) complex is a central player in the regulation of both the initiation of actin polymerization and the organization of the resulting filaments [103]. Members of the Wiscott-Aldrich syndrome protein (WASp) family have been identified as a nucleation-promoting factor (NPF) for the Arp2/3 complex; WASp, N (neural)-WASp, and three WAVE (WASp-family verprolin-homologous protein) isoforms, WAVE 1, 2, and 3, are included in this family [103–105]. They form the nucleation complex of Rho GTPase-mediated actin polymerization by binding to the VCA domain of the WASp proteins at the C-terminus. Functional interaction between Abl and the WASP family has been observed. Namely, WAVE1 translocates to the cell membrane upon PDGF stimulation and is found in a complex containing Abl and other signaling proteins, inducing plasma membrane ruffling [106]. WAVE2 mediates actin reorganization by relaying signals from Rac1 to the Arp2/3 complex, resulting in a lamellipodial protrusion. Abl-dependent phosphorylation of WAVE2 is necessary for this process [107]. On the other hand, phosphorylation of N-WASp at Y256 by Abl is required for *shigella*-promoted actin comet tail elongation [72]. This observation indicates that Abl could regulate N-WASP activity independent of the activation of the Rho GTPase family.

Mena (mammalian-enabled)/VASP (vasodilator-stimulated phosphoprotein) proteins interact with barbed ends of actin-filament and permit its elongation by preventing the capping of the barbed end: the anticapping hypothesis [108]. Indeed, Mena/VASP proteins colocalize to the tips of filopodia and lamellipodia and also at focal adhesion sites [109, 110]. Functional interaction between Ena and Abl was first identified by genetic experiments in flies in which Ena functions as a suppressor of lethality associated with zygotic Abl mutations [111]. Indeed, dAbl can phosphorylate Ena at several tyrosine residues, reducing its ability to bind to SH3-domain-containing proteins [112]. In the mammalian system, Abl can target a single tyrosine residue in Mena [75], but the significance of this event is unclear. However, VASP coimmunoprecipitates with Abl in an adhesion-dependent manner, suggesting the VASP protein complex functions cooperatively with Abl in F-actin reorganization [113].

Cells achieve cell-cell adhesion through cadherin receptors which are linked to the actin cytoskeleton through the catenin complex and are regulated by the Rho GTPase family [114]. E-cadherin interacts with the Arp2/3 complex to promote local actin assembly and lamellipodial protrusion during the formation of early cell-cell adhesion contacts [115]. Abl plays important roles in this intercellular signaling as well. Namely, cell-cell adhesion enhances Abl kinase activity through an as-yet-unknown mechanism, leading to increased CrkII phosphorylation by Abl, and subsequent Rac activation through ELMO [116–118]. Conversely, when Abl kinase activity is lost, it leads to disruption of E-cadherin-based cell-cell contacts [114, 118].

### 5.2. Abi and Crk in Actin Dynamics

Adaptor proteins not only are important for the regulation of Abl as described above, but also regulate the downstream signaling machineries leading to acitn dynamics. Abi and Crk proteins bind Abl and also function as scaffold proteins that permit the assembly of respective multimolecular complexes (Figure 3(b)). This property confers essential but distinct roles to each Abi and Crk protein in Abl-mediated actin dynamics. Indeed, focal adhesion proteins (e.g., paxillin, p130Cas, and Crk) and/or proteins found in filopodial structures (e.g., DOCK180, Abi, and PSTPIP) are included among the targets of Abl downstream of integrins [5]. Abi1 is essential for the stability and integrity of the WAVE complex [119–121]. In the complex, WAVE1 binds to Abi1 through a region within WAVE1 WHD and promotes the localization to the tip of the lamellipodium [73]. On the other hand, Abi1 interacts directly with the WHD domain of WAVE2 and mediates the assembly of a WAVE2-Abi1-Nap1-PIR121 complex. Similar complexes are observed in Abi2 [118] and thought to act as an inhibitory complex for WAVE activity [122, 123]. The Abi1/WAVE2 complex also plays important roles in F-actin dynamics stimulated by colony-stimulating factor-1 in dendritic cells [124] and by T-cell receptor at the T-cell and B-cell contact sites [125]. Regarding this, Abi1-promoted tyrosine phosphorylation at Y150 of WAVE2 by Abl was reported to be important in the regulation of membrane ruffling and dendritic morphogenesis [71]. While the Abi1/WAVE complex plays a role in Rac-dependent membrane dynamics, the Abi1/N-WASP complex, cooperating with cdc42, regulates actin-based vesicular transport, EGFR endocytosis, and EGFR and transferrin receptor cell-surface distribution. Thus, Abi1 is a dual regulator of WAVE and N-WASp activities in the respective processes [126].

Furthermore, Abi1 connects Abl and the substrate. Abi1 binds Mena and VASP through the polyproline region of Abi (Figure 3(b)) and promotes Abl-mediated phosphorylation at Y296 and Y39, respectively [75, 127]. Phosphorylation of VASP abrogates its affinity for Zyxin and subsequent accumulation at focal adhesion sites and modulates leukemic cell adhesion [127]. Furthermore, treatment with imatinib, a selective Abl inhibitor, increases the association of Abl at the lamellipodial cell edge, providing evidence that imatinib leads to the activated conformational change of Abl [128]. Thus, activated Abl presumably faces Mena/VASP and WAVE2 at the lamellipodial tip where Abi1 is localized and phosphorylates these substrates by an Abi1-bridged mechanism [127]. While present in the WAVE complex, Abi3 does not promote Abl-mediated phosphorylation, suggesting differences among Abi family members in the regulation of Abl [129].

Bcr-Abl-positive leukemic cells exhibit abnormalities in cell motility, cell adhesion, and integrin function [130]. These characteristic properties are believed to play critical roles in the progression of CML. Abi1 plays an important role in these processes through direct interaction between its SH3 domain and the C-terminal proline-rich sequences of Bcr-Abl [131]. Knockdown of the expression of Abi1 appeared to suppress Bcr-Abl-induced leukemogenesis *in vivo* and also inhibited Bcr-Abl-stimulated actin cytoskeleton remodeling, MT1-MMP clustering, and cell adhesion and migration *in vitro* [132, 133]. Bcr-Abl subsequently induces tyrosine phosphorylation of Abi1, accompanied by cellular translocation of the Abi1/WAVE2 complex to a site adjacent to the membrane, where an F-actin-enriched structure containing adhesion molecules such as *β*1 integrin, paxillin, and vinculin is assembled. Phosphorylation of Abi1 was reported to enhance the phosphorylation of Mena by Abl, accompanying the enhanced adhesion of leukemic cells and Bcr-Abl transformed cells *in vitro* [90].

Crk, on the other hand, binds and activates Abl kinase as described above, inducing increased tyrosine phosphorylation of p130Cas [76]. The phosphorylated p130Cas then binds the Crk SH2 domain, while DOCK180 binds the Crk SH3 (N) domain (Figure 3(b)), resulting in ternary complex of p130Cas-Crk-DOCK180, which is sufficient to activate Rac1 and localize to focal adhesion sites [134]. The formation of this complex is an important step in the Abl-mediated actin dynamics through Crk. Paxillin contains two Y-x-x-P motifs and also binds the SH2 domain of Crk [135]. Paxillin is a substrate for several tyrosine kinases and functions as a scaffold to organize signal transduction proteins including *α*4 integrin into a large complex at focal adhesion sites [136]. These characteristic properties lead to an accumulation of Crk effectors bound via the Crk SH3 domain at sites of paxillin abundance. C3G was the first Crk SH3 (N) domain-binding protein identified. C3G is a guanine nucleotide releasing Rap1 whose targets have been implicated in cell proliferation, cytoskeletal reorganization during cell adhesion to ECM, and cell-to-cell contact [114, 118].

 While the integrin-induced tyrosine phosphorylation of paxillin and p130Cas [137, 138] is associated with enhanced rates of fibroblast spreading and migration, tyrosine phosphorylation of Crk may negate these effects by disrupting the Abl : Crk : paxillin or Abl : Crk : p130Cas complex [139]. This raises the interesting possibility that Abl may both activate and inactivate cytoskeletal rearrangements at focal adhesion sites during cell spreading and migration and thereby attenuate cell migration by modulating cell adhesion and contractility [140]. Indeed, fibroblasts that are deficient in Abl migrate faster than wild-type controls, and reconstitution with Abl slows these cells [141, 142]. These results also highlight Crk as an important player in Abl-mediated actin dynamics and cell locomotion.

**Figure 5:**
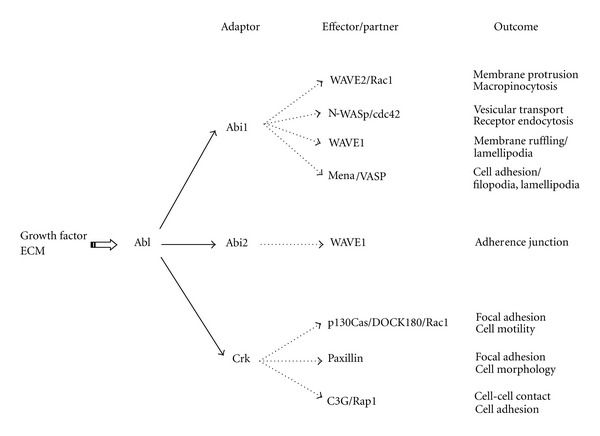
Differential roles of Abi and Crk in signaling pathways of Abl-mediated actin dynamics. Upstream and downstream signaling cascades of Abl-mediated actin dynamics are schematically illustrated (solid arrow). Downstream signaling pathways unique to each member of the Abi and Crk families are also shown together with the respective outcomes (broken arrow).

Finally, upstream signals and downstream signaling cascades of Abl-mediated actin dynamics are schematically summarized in Figure 5 in which signaling pathways unique to each adaptor protein are shown together with the respective outcomes.

## Figures and Tables

**Table 1:** Adaptor proteins for c-Abl in mice.

Adaptor protein	Target domain in Abl	References
c-Crk : CrkI, CrkII	Proline-rich motif	[55–57]
CrkL	Proline-rich motif	[58]
Abi1/E3B1	Proline-rich motif/SH3	[59–61]
Abi2/ArgBP1	Proline-rich motif/SH3	[62, 63]
CAP/Ponsin/SH3P12	Proline-rich motif	[64, 65]
Vinexin	Proline-rich motif	[66]
Nck	Proline-rich motif	[67]
Cbl	Proline-rich motif	[68]
ArgBP2/Sorbs2	Proline-rich motif	[63]

Adaptor proteins that bind Abl and function in Abl-mediated signaling in mouse cells are listed together with the target domain in Abl.
